# Multiple Copies of Tigecycline Gene Cluster *tmexC6D6-toprJ1b* in *Pseudomonas mendocina* in a Swine Farm

**DOI:** 10.3390/antibiotics14050500

**Published:** 2025-05-13

**Authors:** Renjie Wu, Yongliang Che, Longbai Wang, Qiuyong Chen, Bing He, Jingli Qiu, Xuemin Wu, Rujing Chen, Yutao Liu, Lunjiang Zhou

**Affiliations:** 1 Institute of Animal Husbandry and Veterinary Medicine, Fujian Academy of Agricultural Sciences, Fuzhou 350013, China; wurenjie1231@163.com (R.W.); cyl19760810@163.com (Y.C.); wanglongbai@163.com (L.W.); fjchenqiuyong@163.com (Q.C.); he_bing_faas@163.com (B.H.); qiujingli2020@163.com (J.Q.); wxm0593@163.com (X.W.); fjchenrujing@163.com (R.C.); 2 Fujian Animal Diseases Control Technology Development Center, Fuzhou 350013, China

**Keywords:** tigecycline resistance, efflux pump, *tmexC6D6-toprJ1b*, *Pseudomonas*, pig

## Abstract

**Background/Objectives:** The emergence and transmission of the tigecycline resistance efflux pump gene cluster *tmexCD-toprJ* among humans, animals and the environment have posed a serious threat to public health. The objective of this study was to characterize *Pseudomonas* strains carrying multiple copies of *tmexC6D6-toprJ1b* from a pig farm and illustrate the genetic context of *tmexC6D6-toprJ1b* in the NCBI database. **Methods:** The characterization of *Pseudomonas* strains FJFQ21PNM23 and FJFQ21PNM24 was determined by antimicrobial susceptibility testing, whole-genome sequencing, and RT-qPCR. **Results:** The *tmexCD-toprJ*-positive *P. mendocina* strains FJFQ21PNM23 and FJFQ21PNM24 were isolated from nasal swabs in a pig farm. Sequence analysis showed that the two *P. mendocina* strains harbored multiple antimicrobial resistance genes, including tigecycline resistance gene *tmexC6D6-toprJ1b*. WGS analysis indicated that *tmexC6D6-toprJ1b* gene was located on a classical transferable module (*int1*-*int2*-*hp1*-*hp2*-*tnfxB*-*tmexCD-toprJ*) and a multiresistance region in FJFQ21PNM24 and FJFQ21PNM23, respectively. Further analysis revealed that 39 additional *tmexC6D6-toprJ1b* genes in the NCBI database were all identified in *Pseudomonas* spp., and the genetic features of *tmexC6D6-toprJ1b* were summarized into three distinct structures. **Conclusions:** This study is the first to identify and report the tigecycline resistance gene *tmexCD-toprJ* in a swine farm. Our findings summarize the three structures in the genetic context of *tmexC6D6-toprJ1b* and reveal that *Pseudomonas* serves as the only known reservoir of *tmexC6D6-toprJ1b*.

## 1. Introduction

Tigecycline is a semisynthetic glycylcycline, belonging to the third-generation tetracyclines, with a broad spectrum of antimicrobial activity [1]. Notably, tigecycline is recognized as one of the few effective treatments for infections caused by multidrug-resistant (MDR) bacteria [2]. However, the rapid emergence and spread of tigecycline resistance, particularly the plasmid-borne *tet*(X)-encoding flavin-dependent monooxygenase gene and the resistance-nodulation-division (RND) family efflux pump gene cluster *tmexCD-toprJ*, pose a serious threat to public health [1,3]. Since *tmexCD1-toprJ1* was first reported on the plasmid of *Klebsiella pneumoniae*, six *tmexCD-toprJ* variants have been identified in various bacterial species, including *Klebsiella* spp., *Pseudomonas* spp., and *Aeromonas* spp., among others [1,4,5,6]. Notably, *Pseudomonas* is speculated to be an ancestral host of *tmexCD-toprJ* and has played a significant role in the reservoir and transmission of *tmexCD-toprJ* [4,5,7]. Furthermore, TmexCD-TOprJ efflux pump not only mediates tigecycline resistance but also confers multidrug resistance by expelling multiple drugs, such as quinolones, cephalosporins, and aminoglycosides [1,5]. Alarmingly, the co-occurrence of colistin (*mcr*) or carbapenem (e.g., *bla*_NDM_, *bla*_KPC_, *bla*_OXA_) resistance genes in *tmexCD-toprJ*-bearing MDR strains threatens the clinical efficacy of multiple drugs in both human and livestock [8,9,10,11].

To date, six *tmexCD-toprJ* gene clusters have been found in humans, animals, food, and environmental samples [5,12,13,14,15,16]. Although tigecycline has been banned from use in the livestock industry, livestock is the main origin of *tmexCD1-toprJ1* [4,7]. Tetracyclines are the most widely used veterinary antibiotics; they are frequently used to treat infections of the respiratory and digestive tracts in swine farms and serve as feed additives in broiler chicken farming [17,18]. Therefore, the accumulation of tetracycline-resistant pathogens is driven by the selective pressure of this drug, which results in partial tetracycline resistance bacteria showing cross-resistance against tigecycline [7]. Consequently, since *tmexCD1-toprJ1* was first isolated from *K. pneumoniae* in chicken feces in a chicken farm, *tmexCD-toprJ* variants have spread extensively across the chicken food production chain with high positivity rates [1,9,16,19,20]. Moreover, *tmexCD-toprJ* has been sporadically detected in swine slaughterhouses and pork, while it has not been reported in pig farms [1,12,19,20,21]. Therefore, the emergence and dissemination of *tmexCD-toprJ* variants in the pig food production chain remain unclear.

Thus, this study aimed to screen the tigecycline resistance gene cluster *tmexCD-toprJ* among bacteria isolated from a swine farm in Fujian Province, China. In this study, we first detected *tmexCD-toprJ* gene clusters in a pig farm and characterized two *tmexCD-toprJ*-positive *Pseudomonas mendocina* strains. Additionally, this study also characterized the diverse genetic context of *tmexCD6-toprJ1b* in *Pseudomonas* spp.

## 2. Results

### 2.1. Identification of tmexCD-toprJ-Positive Strains

Two swine-origin isolates, FJFQ21PNM23 and FJFQ21PNM24, were identified as *P. mendocina* by matrix-assisted laser desorption ionization–time of flight mass spectrometry (MALDI-TOF MS) (Table 1). Moreover, a PCR screening assay confirmed that both *P. mendocina* strains carried *tmexCD-toprJ*-like gene cluster. Further Sanger sequencing suggested that FJFQ21PNM23 and FJFQ21PNM24 carried a *tmexC6D6*-*toprJ1b* gene cluster (Table 1).

### 2.2. Antimicrobial Susceptibility of tmexC6D6-toprJ1b-Positive P. mendocina

Antimicrobial susceptibility testing suggested that both strains showed resistance to multiple antimicrobial agents, including tetracyclines (MIC ≥ 128 mg/L), doxycycline (MIC = 16 mg/L), florfenicol (MIC > 256 mg/L), chloramphenicol (MIC > 256 mg/L), and trimethoprim–sulfamethoxazole (MIC > 16/304). Additionally, FJFQ21PNM23 exhibited resistance to ciprofloxacin (MIC = 16 mg/L), and FJFQ21PNM24 exhibited resistance to gentamicin (MIC = 16 mg/L) and streptomycin (MIC > 256 mg/L). The two strains were susceptible to cefquinome, cefotaxime, and ceftazidime (Table 2). Notably, the MIC values of tigecycline for FJFQ21PNM23 and FJFQ21PNM24 were 1 and 2, respectively.

### 2.3. Characterization of tmexC6D6-toprJ1b-Positive P. mendocina

To further determine the genetic location of *tmexC6D6*-*toprJ1b* and the antibiotic resistance genes (ARGs) profiles of the two *P. mendocina* strains, the complete genomes of FJFQ21PNM23 and FJFQ21PNM24 were obtained using Illumina and Nanopore sequencing. The sequence analysis suggested that FJFQ21PNM23 harbored a 4.78 Mb chromosome and a 142,064 bp plasmid. Therein, six ARGs were identified on the chromosome, including genes conferring resistance to tetracyclines [*tmexC6D6-toprJ1b* and *tet*(G)], quinolone (*qnrVC1*), chloramphenicol (*floR*), sulfonamide (*sul1*), and aminoglycoside (*aac(6′)-IIa*), while the sulfonamide gene *dfrA1* was located on the plasmid (Table 1). In contrast, FJFQ21PNM24 contained 10 ARGs, namely *tmexC6D6-toprJ1b*, *tet*(G), *aph(6)-Id*, *dfrA1*, *aph(3″)-Ib*, *ant(2″)-Ia*, *dfrA1*, *sul1*, *sul2*, *qnrVC1*, and *floR*, all of which were found on a 4.79 Mb chromosome (Table 1).

### 2.4. Genetic Context of tnfxB6-tmexC6D6-toprJ1b

**Figure 1:**
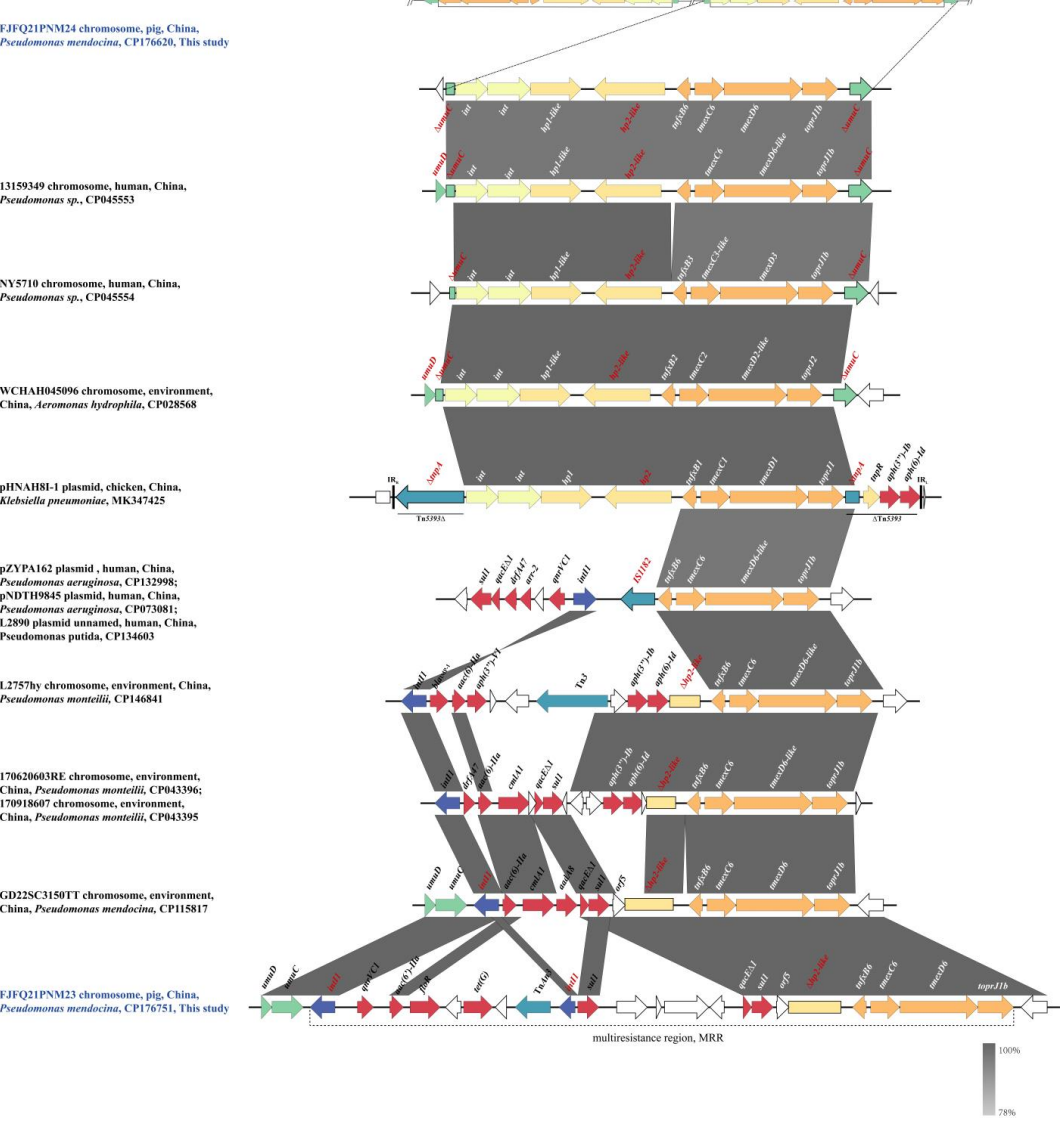
Comparison of the genetic context of *tmexC6D6-toprJ1b* in *Pseudomonas mendocina* FJFQ21PNM23 and FJFQ21PNM24 with those of its similar sequences. The extents and directions of the genes are shown by arrows labeled with the corresponding gene names. The tmexCD-toprJ genes are shown as orange arrows, and other ARGs are depicted by red arrows. Hypothetical protein genes (*hp1*-like and *hp2*-like) and hypothetical integrase genes (*int1* and *int2*) are labeled with dark yellow and light yellow arrows, respectively. The two copies of the classical transferable module (*int1*-*int2*-*hp1*-*hp2*-*tnfxB*-*tmexCD*-*toprJ*) in FJFQ21PNM24 are shown within a box. The MRR in FJFQ21PNM23 is labeled with a dotted line. Other different genes are labeled with corresponding colors. The truncated genes are denoted by the symbol ∆. The plasmid backbone or chromosome is represented by a horizontal black line. The gray shade indicates a nearly 100% homologous region.

**Figure 2:**
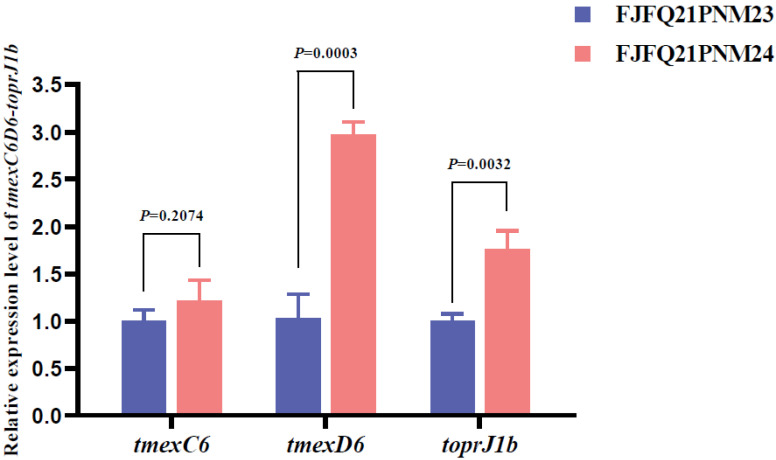
Comparison of the transcriptional expression levels of *tmexC6D6-toprJ1b* gene between *Pseudomonas mendocina* FJFQ21PNM23 and FJFQ21PNM24. The statistical significance of the data was analyzed by Student’s *t*-test.

The function of *tmexC6D6-toprJ1b* and the regulatory effect of TNfxB6 have been reported in a previous study [5]. Here, the genetic structure of chromosomal *tmexC6D6-toprJ1b* located in FJFQ21PNM23 and FJFQ21PNM24 was analyzed. FJFQ21PNM23 harbored one copy of *tmexC6D6-toprJ1b*, whereas FJFQ21PNM24 contained two copies, separated by a genetic distance of approximately 406 kb (Figure 1). As expected, the transcription level of *tmexC6D6-toprJ1b* gene in FJFQ21PNM24 was higher than that in FJFQ21PNM23 (Figure 2), which may account for FJFQ21PNM24 exhibiting two-fold to four-fold higher MICs for tetracyclines compared with FJFQ21PNM23.

Similarly to *tnfxB1-tmexCD1-toprJ1*, *tnfxB2-tmexCD2-toprJ2*, and *tnfxB3-tmexCD3-toprJ1b* gene clusters, two hypothetical protein genes (*hp1*-like and *hp2*-like) and two hypothetical integrase genes (*int1* and *int2*) were located upstream of chromosomal *tnfxB6-tmexC6D6-toprJ1b* in FJFQ21PNM24 (Figure 1), forming a classical transferable module (*int1*-*int2*-*hp1*-*hp2*-*tnfxB*-*tmexCD-toprJ*). In addition to *tnfxB1-tmexCD1-toprJ1*, the transferable module was found to specifically insert into the *umuC*-like gene. An identical *tnfxB6-tmexC6D6-toprJ1b*-carrying module region was inserted into the *umuC*-like gene on the chromosome of *Pseudomonas* sp. strain 13,159,349 (CP045553.1). In addition, this module was also found on the plasmid of *Pseudomonas aeruginosa* S201409-209 (CP131786.1) and S201405-249 (CP131793.1), as well as on the chromosomes of *Pseudomonas juntendi* L4008hy (CP146690.1) and *Pseudomonas alcaligenes* KAM426 (AP024354.1) (Figure S1). Unlike the genetic context of *tnfxB*-*tmexCD-toprJ* in the classical transferable module, *tnfxB6-tmexC6D6-toprJ1b* was inserted into a 28,302 bp multiresistance region (MRR) upstream of the *umuC* and *umuD* genes in FJFQ21PNM23 (Figure 1) [15]. The MRR carried *qnrVC1*, *aac(6′)-IIa*, *floR*, *tet*(G), *tmexC6D6-toprJ1b*, and two copies of *sul1* resistance genes. Interestingly, all chromosomal resistance genes in FJFQ21PNM23 were located within this MRR. Further genomic analysis showed that the 10,848 bp structure *qacE∆1*-*sul1*-*orf5*-*∆hp2*-like-*tnfxB6-tmexC6D6-toprJ1b* in FJFQ21PNM23 was identical to the corresponding region of the first reported *tnfxB6-tmexC6D6-toprJ1b*-positive *P. mendocina* GD22SC3150TT chromosome (CP115817.1) and *Pseudomonas stutzeri* ZDHY95 chromosome (CP063358.1) (Figure S2).

The Nucleotide Basic Local Alignment Search Tool (BLASTn) analysis results revealed that 39 additional *tmexC6D6-toprJ1b* or *tmexC6D6-toprJ1b*-like gene clusters (>99.60% nucleotide identity) originating from different *Pseudomonas* species exhibited diverse genetic environments of *tmexC6D6-toprJ1b* (Table S2). The genomic context analysis suggested that the highly homologous structure *aph(3″)-Ib*-*aph(6)-Id*-*∆hp2*-like-*tnfxB6-tmexC6D6-toprJ1b* was located on the chromosomal *tnfxB6-tmexC6D6-toprJ1b*-carrying MRRs of *Pseudomonas kurunegalensis* NY4817 (CP131921.1), *P. aeruginosa* ZM21 (CP141683.1), *Pseudomonas* sp. BJP69 (CP041933), *Pseudomonas monteilii* L2757hy (CP146841.1), *P. monteilii* 170620603RE (CP043396.1), and *P. monteilii* 170918607 (CP043395.1) (Figure S2). Of note, the *∆hp-2*-like gene in the NY4817-like strains showed 99.91–100% identity but only 41–59% coverage compared to the corresponding gene in FJFQ21PNM24 and FJFQ21PNM23 (Table S2). In addition to the classical structure (*int1*-*int2*-*hp1*-*hp2*) and a part of the *hp2* gene, *tnfxB6-tmexC6D6-toprJ1b* was adjacent to IS*1182* (Figure S1), where two copies of IS*1182* flanked the *tnfxB6-tmexC6D6-toprJ1b* gene cluster on the plasmid (CP084322.1) or chromosome (CP061777.1 and CP061779.1) of *P. aeruginosa*. Notably, the structures *sul1*-*qacE∆1*-*drfA47*-*arr-2*-*qnrVC1* and IS*1182*-*tnfxB6-tmexC6D6-toprJ1b* formed an MRR that was found on the plasmids of *P. aeruginosa* (CP132998.1, CP073081.1, and CP095921.1) and *Pseudomonas putida* (CP134603.1) (Figure S1).

## 3. Discussion

Unlike the prevalence of *tmexCD1-toprJ1*, *tmexCD2-toprJ2*, and *tmexCD3-toprJ1b* among *tmexCD-toprJ* gene clusters, *tmexC6D6-toprJ1b* has rarely been reported [4]. Here, we identified two *P. mendocina* strains carrying chromosomal *tmexC6D6-toprJ1b*, which originated from a pig farm in China. The *tmexCD-toprJ* gene cluster has been reported in humans, environmental samples (e.g., sewage from urban areas, hospitals, and food markets, farm markets, and river water), food products (e.g., vegetables, fish meat, chicken meat), and animals (e.g., chickens, migratory birds, fish, ducks, flies) [1,5,6,20,22,23,24,25,26,27]. Despite the widespread dissemination of the *tmexCD-toprJ* gene cluster among various sources in China, it has been sporadically reported in swine and has only been found in swine feces from slaughterhouses and retail pork (Table S3) [1,12,19,20]. It is noteworthy that this study is the first to report the *tmexCD-toprJ* gene cluster isolated from nasal swabs from swine in a pig farm.

The *tmexC6D6-toprJ1b* was first detected in *P. mendocina* of environmental origin, and its variant *tmexC6D6.2-toprJ1b* was reported in *P. aeruginosa* from a retail chicken sample [5,11]. Subsequently, this study first identified multiple copies of *tmexC6D6-toprJ1b* in *Pseudomonas* spp. However, we did not detect multiple copies of *tmexC6D6-toprJ1b* in 39 additional *tmexC6D6-toprJ1b*-like-positive strains from the NCBI database (Table S2). In contrast, multiple copies of *tmexCD2-toprJ2*-like gene were found on the chromosome of *Aeromonas* spp. [26]. Unlike the first reported *tmexC6D6-toprJ1b*-carrying strain *P. mendocina* (MIC = 8), the MICs of tigecycline in FJFQ21PNM23 (MIC = 1 mg/L) and FJFQ21PNM24 (MIC = 2 mg/L) were lower. *P. mendocina* GD22SC3150TT (MIC = 8) was isolated using an agar plate supplemented with 4 mg/L tigecycline, whereas *P. mendocina* FJFQ21PNM23 (MIC = 1 mg/L) and FJFQ21PNM24 (MIC = 2 mg/L) were randomly obtained using agar plates without antibiotics, which may have resulted in the differences in the MICs of tigecycline of *P. mendocina* between the two studies [5]. In addition to the intrinsic resistance of *P. aeruginosa* to tigecycline, the MICs of tigecycline for other *Pseudomonas* strains carrying *tmexCD-toprJ* ranged from 1 to 16 mg/L [5,7,24,28].

To date, *K. pneumoniae* and *Pseudomonas* spp. have been the predominant *tmexCD-toprJ*-bearing strains, while *tmexCD-toprJ* gene was also found in *Aeromonas* spp., *Raoultella ornithinolytica*, *Oceanimonas* sp., *Proteus* spp., *Citrobacter youngae*, and other *Enterobacteriaceae* [4,10,13,14,15,26]. Although *Escherichia coli* carrying *tmexCD-toprJ* was found in the NCBI database using bioinformatics analysis [4,8], we supposed that *E. coli* may harbor *tmexCD-toprJ* spread among the intestinal tract. The BLASTn analysis revealed that *tmexC6D6-toprJ1b* or *tmexC6D6-toprJ1b*-like gene clusters in our study and the NCBI database were all located on the plasmids and chromosomes of *Pseudomonas* spp. (Table S2), indicating that *Pseudomonas* is the only known repository of *tmexC6D6-toprJ1b*. Of these 41 *Pseudomonas* spp., *tmexC6D6-toprJ1b* or *tmexC6D6-toprJ1b*-like genes were predominantly identified in *P. aeruginosa* (n = 16), while *P. stutzeri* (n = 5), *P. putida* (n = 4), and *P. monteilii* (n = 3) also acted as reservoirs of *tmexC6D6-toprJ1b* (Table S2). Previous studies have showed that chromosomal *mexCD-oprJ* in *P. aeruginosa* serves as a potential ancestral source of tigecycline efflux pump *tmexCD-toprJ* clusters [4,29]. In addition to *tmexCD1-toprJ1* and *tmexCD1-toprJ2*, other *tmexCD-toprJ* were mostly found in *Pseudomonas* [4,5,7,13,28]. Consequently, we surmise that *Pseudomonas* spp. play a vital role in the reservoir and transmission of *tmexCD-toprJ* gene clusters.

The *tmexCD1-toprJ1* was first found in 2020 in China; since then, *tmexCD-toprJ* gene clusters have been globally reported, mainly in China and some Asian countries [1,4]. The BLASTn analysis revealed that *tmexC6D6-toprJ1b* or *tmexC6D6-toprJ1b*-like genes were isolated from seven countries, including China, Japan, Lebanon, Pakistan, Spain, Poland, and Brazil (Table S2). The chromosomal *tmexC6D6-toprJ1b* of FJFQ21PNM24 was identified on a similar genetic module (*int1*-*int2*-*hp1*-*hp2*-*tnfxB*-*tmexCD-toprJ*) to those found in the *tmexCD1-toprJ1*, *tmexCD2-toprJ2*, and *tmexCD3-toprJ1b* gene clusters (Figure 1). This genetic structure carrying *tmexC6D6-toprJ1b* was found in *P. mendocina*, *P. juntendi*, *P. alcaligenes*, *P. aeruginosa*, and *Pseudomonas* sp. originating from humans and animals, revealing that it can promote the transfer of *tmexC6D6-toprJ1b* (Figure S1). Although a previous study summarized seven types of genomic environments of *tmexCD-toprJ* in *Pseudomonas* spp. [29], the genetic context of *tmexC6D6-toprJ1b* can be simplified into three structures. In addition to the classical transfer module structure, the other two structures are characterized by an insertion sequence (IS) or *∆hp2*-like gene combined with a gene cassette carrying multiple antibiotic genes, located upstream of *tmexC6D6-toprJ1b* to form an MRR. Further analysis illustrated that the structure (resistances-*intI1*-IS-*tnfxB6*-*tmexC6D6-toprJ1b*) was predominantly found in *P. aeruginosa*, while the structure (*intI1*-resistances-*∆hp2*-like-*tnfxB6*-*tmexC6D6-toprJ1b*) was identified in various *Pseudomonas* spp. (Figures S1 and S2). Notably, *tmexC6D6-toprJ1b*-bearing *Pseudomonas* strains carried not only multidrug resistance efflux pump TmexC6D6-TOprJ1 but also multiple antibiotic resistance genes, such as carbapenem (*bla*_OXA-246_, *bla*_IMP-1_, *bla*_VIM-2_, *bla*_IMP-15_), aminoglycoside [*aac(6′)-IIa*, *aph(3″)-Ib*, *aph(6)-Id*], sulfonamide (*dfrA47*, *sul1*, *dfrB1*), and other resistance genes. This highlights the need for vigilance regarding the emergence and spread of *tmexC6D6-toprJ1b* among *Pseudomonas*. Given the limited reports on *tmexC6D6-toprJ1b* [5,11], future studies should also prioritize monitoring *tmexC1D1-toprJ1*, *tmexC2D2-toprJ2*, and *tmexC3D3-toprJ1b* in swine-origin bacteria.

## 4. Materials and Methods

### 4.1. Bacterial Strains

A total of 77 isolates were collected from one lung sample and 19 nasal swabs from a swine farm in Fujian Province, China, in September 2021. Among them, the strains FJFQ21PNM23 and FJFQ21PNM24 were isolated from nasal swabs. These isolated strains were collected with tryptic soy agar (TSA) (OXOID, Basingstoke, UK) supplemented with 5% sterile defibrillated equine blood (Zhengzhou Jiulong Biotechnology, Zhengzhou, China).

Bacterial species identification was performed using MALDI-TOF MS (Bruker Daltonics, Bremen, Germany). An isolated colony was selected as a sample, which was transferred onto the target plate. A matrix was added to the sample on the plate, and the MALDI-TOF spectrum was automatically generated by the software.

Subsequently, PCR was performed to screen for *tmexCD-toprJ*-like gene clusters using previously described primers (Table S1) [1]. PCR was performed using the GoTaq^®^ Green Master Mix (Promega, Beijing, China) on the VeritiPro^TM^ thermal cycle (Thermo Fisher Scientific, Waltham, MA, USA). The amplification was performed under the following conditions: 95 °C for 2 min; 30 cycles of 95 °C, 30 s; 60 °C, 30 s; 72 °C, 1 min, followed by the final extension of 72 °C at 5 min. The positive products were subjected to Sanger sequencing (TsingKe Biological Technology, Beijing, China), and the obtained sequences were verified by the National Center for Biotechnology Information (NCBI) BLAST (http://blast.ncbi.nlm.nih.gov/Blast.cgi, accessed on 12 June 2024).

### 4.2. Antimicrobial Susceptibility Testing

The minimum inhibitory concentrations (MICs) of *tmexCD-toprJ*-positive strains against 13 antimicrobial agents were determined using broth microdilution (for tigecycline) and agar dilution methods (for minocycline, doxycycline, tetracycline, cefquinome, cefotaxime, ceftazidime, florfenicol, chloramphenicol, gentamicin, streptomycin, ciprofloxacin, and trimethoprim–sulfamethoxazole) based on the Clinical and Laboratory Standard Institute guidelines (CLSI, M100-S31) [30]. Mueller–Hinton (MH) agar (Hope Biotechnology, Qingdao, China) was used in the agar dilution method. The MH broth (Hope Biotechnology, Qingdao, China) and a 96-well cell culture plate (Beijing Labgic Technology, Beijing, China) were used in the broth microdilution method. *Escherichia coli* ATCC 25922 served as the quality control strain. The MICs were interpreted in accordance with the CLSI and European Committee on Antimicrobial Susceptibility Testing (EUCAST, version 9.0) [31] guidelines (for tigecycline).

### 4.3. Whole-Genome Sequencing (WGS) Analysis

The genomic DNA of *tmexC6D6-toprJ1b*-positive strains was extracted using the Hipure Bacterial DNA Kit (Magen, Guangzhou, China), according to the product manual. The whole-genome sequencing of two *P. mendocina* strains, FJFQ21PNM23 and FJFQ21PNM24, was performed using the Illumina NovaSeq and Oxford Nanopore MinION platforms (Beijing Novogene Technology, Beijing, China). The de novo assembly was obtained using SPAdes, and the complete sequences were generated through hybrid assembly with Unicycler [32,33]. Antimicrobial resistance genes (ARGs) were analyzed using Resfinder from the Center for Genomic Epidemiology server (http://genepi.food.dtu.dk/resfinder, accessed on 12 June 2024) [34]. The genomic environmental analysis of *tmexC6D6-toprJ1b* was performed by Easyfig [35]. To compare the genetic contexts of *tmexC6D6-toprJ1b*, our study collected and summarized the characteristics of an additional 39 strains carrying *tmexC6D6-toprJ1b* from the NCBI database (Table S2).

### 4.4. RNA Extraction, cDNA Synthesis, and RT-qPCR

The total RNA of *tmexC6D6-toprJ1b*-positive strains was extracted using the TransZol Up Plus RNA Kit (TransGen Biotech, Beijing, China), according to the product manual. The quality of the RNA was assessed by agarose gel electrophoresis and quantified by Nanodrop (Thermo Fisher Scientific, Waltham, MA, USA) [36]. Subsequently, cDNA was synthesized using TransScript One-Step gDNA Removal and cDNA Synthesis SuperMix (TransGen Biotech, Beijing, China). qPCR was performed using the PerfectStart Green qPCR SuperMix (TransGen Biotech, Beijing, China) on the LightCycle^®^ 96 real-time PCR detection system (Roche, Basel, Switzerland). The cq value was analyzed by the software LightCycle^®^ 96 SW 1.1, according to the product manual. The relative transcriptional expression levels of *tmexC6*, *tmexD6*, and *toprJ1b* were determined using the 2^−ΔΔCt^ method, with the 16S rRNA gene used as a reference gene [37]. The data on the transcriptional expression levels of *tmexC6D6-toprJ1b* gene passed the normality test, as analyzed by the Shapiro–Wilk test. The statistical significance of the data was further analyzed by Student’s *t*-test. The qPCR primers are listed in Table S1.

### 4.5. Nucleotide Sequence Accession Numbers

The complete nucleotide sequences of the chromosomes of strains FJFQ21PNM23 and FJFQ21PNM24 were deposited in GenBank under the accession numbers CP176751.1 and CP176620.1, respectively.

## 5. Conclusions

In summary, we report, for the first time, the tigecycline resistance gene cluster *tmexCD-toprJ* in nasal samples from a pig farm. Our study identified multiple copies of *tmexC6D6-toprJ1b* and summarized the three genetic contexts of *tmexC6D6-toprJ1b* in *Pseudomonas*. As the only known reservoir of *tmexC6D6-toprJ1b* and a potential ancestral origin of *tmexCD-toprJ*, greater attention and surveillance should be directed toward the emergence and transmission of *tmexCD-toprJ* in *Pseudomonas* spp.

## Figures and Tables

**Table 1:** Characterization of the *tmexC6D6-toprJ1b*-positive *P. mendocina* strains in this study.

Strain	Species	Source	Plasmid or Chromosome	Size (bp)	Resistance Genes
FJFQ21PNM23	*Pseudomonas mendocina*	Nasal swabs of swine	Chromosome	4,779,589	*aac(6′)-IIa*, *qnrVC1*, *floR*, *sul1*, *tet*(G), *tmexC6D6-toprJ1b*
			Plasmid pFJFQ21PNM23 ^a^	142,064	*dfrA1*
FJFQ21PNM24	*Pseudomonas mendocina*	Nasal swabs of swine	Chromosome	4,785,273	*aph(6)-Id*, *dfrA1*, *aph(3″)-Ib*, *ant(2″)-Ia*, *qnrVC1*, *floR*, *sul1*, *sul2*, *tet*(G), *tmexC6D6-toprJ1b*

^a^ The replicon type of pFJFQ21PNM23 was unknown according to WGS analysis.

**Table 2:** MIC (mg/L) values of 13 different antimicrobials for two *P. mendocina* isolates FJFQ23PNM23 and FJFQ23PNM24 carrying *tmexC6D6-toprJ1b*.

	MIC of Drug ^a^												
Strain	TIG	MIN	DOX	TET	CQM	CTX	CAZ	FFC	CHL	GEN	STR	CIP	SXT
FJFQ21PNM23	1	1	16	128	4	2	1	>256	>256	1	4	8	>16/304
FJFQ21PNM24	2	4	16	256	4	2	4	>256	>256	16	>256	2	>16/304

^a^ Abbreviations: TIG, tigecycline; MIN, minocycline; DOX, doxycycline; TET, tetracycline; CQM, cefquinome; CTX, cefotaxime; CAZ, ceftazidime; FFC, florfenicol; CHL, chloramphenicol; GEN, gentamicin; STR, streptomycin; CIP, ciprofloxacin; SXT, trimethoprim–sulfamethoxazole.

## Data Availability

The original contributions presented in this study are included in the article/Supplementary Materials. Further inquiries can be directed to the corresponding author.

## Supplementary Materials

The following supporting information can be download at: https://www.mdpi.com/article/10.3390/antibiotics14050500/s1. Figure S1: Comparison of the genetic context of *tmexC6D6-toprJ1b* in *Pseudomonas mendocina* FJFQ21PNM24 with those of similar sequences carrying *tmexC6D6-toprJ1b*. The *tmexCD-toprJ* genes are shown as orange arrows, while other ARGs are depicted by red arrows. Other different genes are labeled with corresponding colors. The truncated genes are denoted by the symbol *∆*. The plasmid backbone or chromosome is represented by a horizontal black line. The gray shade indicates the nearly 100% homologous region. Figure S2: Comparison of the genetic context of *tmexC6D6-toprJ1b* in *Pseudomonas mendocina* FJFQ21PNM23 with those of similar sequences carrying *tmexC6D6-toprJ1b*. The *tmexCD-toprJ* genes are shown as orange arrows, and other ARGs are depicted by red arrows. Other different genes are labeled with corresponding colors. The truncated genes are denoted by the symbol ∆. The plasmid backbone or chromosome is represented by a horizontal black line. The gray shade indicates the nearly 100% homologous region. Table S1: Primers used in this study; Table S2: *tmexC6D6-toprJ1b* and *tmexC6D6-toprJ1b*-like gene clusters found in GenBank that all originated from *Pseudomonas* spp.; Table S3: The information for *tmexC6D6-toprJ1b* and *tmexC6D6-toprJ1b*-like gene clusters of pig origin. References [1,12,19,20,21,26] are cited in the supplementary materials.

## References

[1] Lv L., Wan M., Wang C., Gao X., Yang Q., Partridge S.R., Wang Y., Zong Z., Doi Y., Shen J. (2020). Emergence of a Plasmid-Encoded Resistance-Nodulation-Division Efflux Pump Conferring Resistance to Multiple Drugs, Including Tigecycline, in Klebsiella pneumoniae. mBio.

[2] Tasina E., Haidich A.B., Kokkali S., Arvanitidou M. (2011). Efficacy and safety of tigecycline for the treatment of infectious diseases: A meta-analysis. Lancet Infect. Dis..

[3] He T., Wang R., Liu D., Walsh T.R., Zhang R., Lv Y., Ke Y., Ji Q., Wei R., Liu Z. (2019). Emergence of plasmid-mediated high-level tigecycline resistance genes in animals and humans. Nat. Microbiol..

[4] Wang C., Yang J., Xu Z., Lv L., Chen S., Hong M., Liu J.H. (2024). Promoter regulatory mode evolution enhances the high multidrug resistance of tmexCD1-toprJ1. mBio.

[5] Wang C.Z., Gao X., Liang X.H., Lv L.C., Lu L.T., Yue C., Cui X.X., Yang K.E., Lu D., Liu J.H. (2023). Pseudomonas Acts as a Reservoir of Novel Tigecycline Resistance Efflux Pump tmexC6D6-toprJ1b and tmexCD-toprJ Variants. Microbiol. Spectr..

[6] Wu Y., Dong N., Cai C., Zeng Y., Lu J., Liu C., Wang H., Zhang Y., Huang L., Zhai W. (2023). *Aeromonas* spp. from hospital sewage act as a reservoir of genes resistant to last-line antibiotics. Drug Resist. Updat..

[7] Wang C., Gao X., Zhang X., Yue C., Lv L., Lu L., Liu J.H. (2025). Emergence of two novel tmexCD-toprJ subtypes mediating tigecycline resistance in the megaplasmids from Pseudomonas putida. Microbiol. Res..

[8] Wu Y., Zhuang Y., Wu C., Jia H., He F., Ruan Z. (2024). Global emergence of Gram-negative bacteria carrying the mobilised RND-type efflux pump gene cluster tmexCD-toprJ variants. Lancet Microbe.

[9] Sun S., Gao H., Liu Y., Jin L., Wang R., Wang X., Wang Q., Yin Y., Zhang Y., Wang H. (2020). Co-existence of a novel plasmid-mediated efflux pump with colistin resistance gene mcr in one plasmid confers transferable multidrug resistance in Klebsiella pneumoniae. Emerg. Microbes Infect..

[10] Wu Y., Dong N., Cai C., Zhang R., Chen S. (2023). Hospital wastewater as a reservoir for the tigecycline resistance gene cluster tmexCD-toprJ. Lancet Microbe.

[11] Wu X., Chen G., Wang P., Yang L., Wu Y., Wu G., Li H., Shao B. (2025). Co-existence of a novel RND efflux pump tmexC6D6.2-toprJ1b and bla(OXA-4) in the extensively drug-resistant Pseudomonas aeruginosa ST233 clone. Int. J. Food Microbiol..

[12] Peng K., Wang Q., Yin Y., Li Y., Liu Y., Wang M., Qin S., Wang Z., Li R. (2021). Plasmids Shape the Current Prevalence of tmexCD1-toprJ1 among Klebsiella pneumoniae in Food Production Chains. mSystems.

[13] Wang C.Z., Gao X., Lv L.C., Cai Z.P., Yang J., Liu J.H. (2021). Novel tigecycline resistance gene cluster tnfxB3-tmexCD3-toprJ1b in Proteus spp. and Pseudomonas aeruginosa, co-existing with tet(X6) on an SXT/R391 integrative and conjugative element. J. Antimicrob. Chemother..

[14] Wang C.Z., Gao X., Yang Q.W., Lv L.C., Wan M., Yang J., Cai Z.P., Liu J.H. (2021). A Novel Transferable Resistance-Nodulation-Division Pump Gene Cluster, tmexCD2-toprJ2, Confers Tigecycline Resistance in *Raoultella ornithinolytica*. Antimicrob. Agents Chemother..

[15] Wang J., Jiang Y., Lu M.J., Wang Z.Y., Jiao X. (2023). Emergence of a novel tigecycline resistance gene cluster tmexC3-tmexD5-toprJ2b in *Oceanimonas* sp. from chicken, China. J. Antimicrob. Chemother..

[16] Gao X., Wang C., Lv L., He X., Cai Z., He W., Li T., Liu J.H. (2022). Emergence of a Novel Plasmid-Mediated Tigecycline Resistance Gene Cluster, tmexCD4-toprJ4, in *Klebsiella quasipneumoniae* and *Enterobacter roggenkampii*. Microbiol. Spectr..

[17] Krishnasamy V., Otte J., Silbergeld E. (2015). Antimicrobial use in Chinese swine and broiler poultry production. Antimicrob. Resist. Infect. Control.

[18] Mulchandani R., Wang Y., Gilbert M., Van Boeckel T.P. (2023). Global trends in antimicrobial use in food-producing animals: 2020 to 2030. PLoS Glob Public Health.

[19] Peng K., Li Y., Wang Q., Yang P., Wang Z., Li R. (2023). Integrative conjugative elements mediate the high prevalence of tmexCD3-toprJ1b in Proteus spp. of animal source. mSystems.

[20] Sun L., Wang H., Meng N., Wang Z., Li G., Jiao X., Wang J. (2023). Distribution and Spread of the Mobilized RND Efflux Pump Gene Cluster tmexCD-toprJ in Klebsiella pneumoniae from Different Sources. Microbiol. Spectr..

[21] Wang Q., Peng K., Liu Y., Xiao X., Wang Z., Li R. (2021). Characterization of TMexCD3-TOprJ3, an RND-Type Efflux System Conferring Resistance to Tigecycline in Proteus mirabilis, and Its Associated Integrative Conjugative Element. Antimicrob. Agents Chemother..

[22] Dong N., Zeng Y., Wang Y., Liu C., Lu J., Cai C., Liu X., Chen Y., Wu Y., Fang Y. (2022). Distribution and spread of the mobilised RND efflux pump gene cluster tmexCD-toprJ in clinical Gram-negative bacteria: A molecular epidemiological study. Lancet Microbe.

[23] Hirabayashi A., Dao T.D., Takemura T., Hasebe F., Trang L.T., Thanh N.H., Tran H.H., Shibayama K., Kasuga I., Suzuki M. (2021). A Transferable IncC-IncX3 Hybrid Plasmid Cocarrying bla(NDM-4), tet(X), and tmexCD3-toprJ3 Confers Resistance to Carbapenem and Tigecycline. mSphere.

[24] Li R., Peng K., Xiao X., Liu Y., Peng D., Wang Z. (2021). Emergence of a multidrug resistance efflux pump with carbapenem resistance gene blaVIM-2 in a Pseudomonas putida megaplasmid of migratory bird origin. J. Antimicrob. Chemother..

[25] Wan M., Gao X., Lv L., Cai Z., Liu J.H. (2021). IS26 Mediates the Acquisition of Tigecycline Resistance Gene Cluster tmexCD1-toprJ1 by IncHI1B-FIB Plasmids in *Klebsiella pneumoniae* and *Klebsiella quasipneumoniae* from Food Market Sewage. Antimicrob. Agents Chemother..

[26] Wang C.Z., Gao X., Tu J.Y., Lv L.C., Pu W.X., He X.T., Jiao Y.X., Deng Y.T., Liu J.H. (2022). Multiple Copies of Mobile Tigecycline Resistance Efflux Pump Gene Cluster tmexC2D2.2-toprJ2 Identified in Chromosome of *Aeromonas* spp.. Microbiol. Spectr..

[27] Yan Z., Wang P., Wang H., Zhang J., Zhang Y., Wu Y., Zhou H., Li Y., Shen Z., Chen G. (2024). Emergence and genomic epidemiology of tigecycline resistant bacteria of fly origin across urban and rural China. Environ. Int..

[28] Zhu J., Lv J., Zhu Z., Wang T., Xie X., Zhang H., Chen L., Du H. (2023). Identification of TMexCD-TOprJ-producing carbapenem-resistant Gram-negative bacteria from hospital sewage. Drug Resist. Updat..

[29] Peng K., Liu Y.X., Sun X., Wang Q., Song L., Wang Z., Li R. (2024). Large-scale bacterial genomic and metagenomic analysis reveals Pseudomonas aeruginosa as potential ancestral source of tigecycline resistance gene cluster tmexCD-toprJ. Microbiol. Res..

[30] Clinical and Laboratory Standards Institute (CLSI) (2021). Performance Standards for Antimicrobial Susceptibility Testing—31th Edition: M100.

[31] EUCAST (2019). Breakpoint Tables for Interpretation of MICs and Zone Diameters, Version 9.0.

[32] Bankevich A., Nurk S., Antipov D., Gurevich A.A., Dvorkin M., Kulikov A.S., Lesin V.M., Nikolenko S.I., Pham S., Prjibelski A.D. (2012). SPAdes: A new genome assembly algorithm and its applications to single-cell sequencing. J. Comput. Biol..

[33] Wick R.R., Judd L.M., Gorrie C.L., Holt K.E. (2017). Unicycler: Resolving bacterial genome assemblies from short and long sequencing reads. PLoS Comput. Biol..

[34] Zankari E., Hasman H., Cosentino S., Vestergaard M., Rasmussen S., Lund O., Aarestrup F.M., Larsen M.V. (2012). Identification of acquired antimicrobial resistance genes. J. Antimicrob. Chemother..

[35] Sullivan M.J., Petty N.K., Beatson S.A. (2011). Easyfig: A genome comparison visualizer. Bioinformatics.

[36] Yang J., Wu R., Xia Q., Yu J., Yi L.X., Huang Y., Deng M., He W.Y., Bai Y., Lv L. (2023). The evolution of infectious transmission promotes the persistence of mcr-1 plasmids. mBio.

[37] Livak K.J., Schmittgen T.D. (2001). Analysis of relative gene expression data using real-time quantitative PCR and the 2(-Delta Delta C(T)) Method. Methods.

