# Supplementary material for: Multiple Copies of Tigecycline Gene Cluster tmexC6D6-toprJ1b in Pseudomonas mendocina in a Swine Farm
Source: Antibiotics (Basel). 2025 May 13;14(5):500. doi: 10.3390/antibiotics14050500 (PMC12108250; doi:10.3390/antibiotics14050500)
Supplement: Supplementary file 1 [file antibiotics-14-00500-s001.zip › antibiotics-3531366-supplementary Tables-xml.pdf]

Supplementary Materials

Table S1 Primers used in this study

Purpose	Primer name	Sequence (5′ to 3′)	Reference
Detection of *tmexCD-toprJ*-like gene clusters	tmexC-F	TTCCGTGATCTCCTGTTTG	[1]
	tmexC-R	GATGGCGTTCTGGTTGAG	
	tmexD-F	CAGCCAGGACTACAACTTC	[1]
	tmexD-R	TAGAGGAACTTCGGATTGC	
To measure the transcriptional expression level of *tmexC6D6-toprJ1b* by qPCR	q-tmexC6-F	CACGCTGCTGTTCACTGATATCT	This study
	q-tmexC6-R	TACATACCCGGCAGCAACAC	
	q-tmexD6-F	TGAGGAGCTGGAAGAGTTTCTGA	This study
	q-tmexD6-R	CGGCGACTGCGAAGAATC	
	q-toprJ1b-F	GTGGACGACTACCTGCGTTATCT	This study
	q-toprJ1b-R	GGCCATCGGTTCAGTTTGG	
	q-16sRNA-F	TGTAGCGGTGAAATGCGTAGA	[2]
	q-16sRNA-R	CACCTGAGCGTCAGTCTTCGT	

Table S2 *tmexC6D6-toprJ1b* and *tmexC6D6-toprJ1b*-like gene clusters found in GenBank that all originated from *Pseudomonas* spp.

Strain name	GenBank accession number	Nucleotide identity compared with *tmexC6D6-toprJ1b*	Nucleotide identity and coverage compared with *hp-2*-like gene of FJ21PNM24	Species	Location of *tmexC6D6-toprJ1b* gene cluster	source	country
GD22SC3150TT	CP115817.1	100%	99.95% and 64%[a]	*Pseudomonas mendocina*	Chromosome	Farm market's sample (Environment)	China
SB-1	CP088004.1	100%	—	*Pseudomonas stutzeri*	Chromosome	Sediment (Environment)	China
S201405-249	CP131793.1	100%	100% and 100%	*Pseudomonas aeruginosa*	Plasmid	Blood (Human)	China
ZDHY95	CP063358.1	100%	99.95% and 64%[a]	*Pseudomonas stutzeri*	Chromosome	Cerebrospinal fluid (Human)	China
L4008hy	CP146690.1	100%	100% and 100%	*Pseudomonas juntendi*	Chromosome	Stool (Human)	China
S201409-209	CP131786.1	100%	100% and 100%	*Pseudomonas aeruginosa*	Plasmid	Blood (Human)	China
MyJU62	DAFQDY010000193.1	100%	100% and 64%	*Pseudomonas aeruginosa*	Unknown	Human	Myanmar
13159349	CP045553.1	99.98%	100% and 100%	*Pseudomonas* sp.	Chromosome	Abdominal fluid (Human)	China

NDTH9845	CP073081.1	99.83%	—	*Pseudomonas aeruginosa*	Plasmid	Human	China
ZYPA162	CP132998.1	99.83%	—	*Pseudomonas aeruginosa*	Plasmid	Sputum (Human)	China
HS18-89	CP084322.1	99.83%	—	*Pseudomonas aeruginosa*	Plasmid	Urine (Human)	China
3860P	CP151176.1	99.83%	—	*Pseudomonas aeruginosa*	Plasmid	Skin (Human)	China
GD04132	CP104579.1	99.83%	—	*Pseudomonas oleovorans*	Chromosome	Surface swab (Environment)	Pakistan
ZM23	CP113432.1	99.83%	—	*Pseudomonas triclosanedens*	Chromosome	Activated sludge (Environment)	China
L2757hy	CP146841.1	99.83%	100% and 41%	*Pseudomonas monteilii*	Chromosome	Stool (Human)	China
L2890	CP134603.1	99.83%	—	*Pseudomonas putida*	Plasmid	Stool (Human)	China
KAM426	AP024354.1	99.83%	100% and 100%	*Pseudomonas alcaligenes*	Chromosome	Unknown	Japan
BJP69	CP041933.1	99.83%	99.91% and 41%	*Pseudomonas* sp.	Chromosome	Sputum specimen (Human)	China
AR19640	CP095921.1	99.83%	—	*Pseudomonas aeruginosa*	Plasmid	Rectal swab (Human)	China
AHSWHJXPP1	CP120969.1	99.83%	100% and 41%	*Pseudomonas putida*	Chromosome	Urine (Human)	China
SRMPA3860	CP132994.1	99.83%	—	*Pseudomonas aeruginosa*	Plasmid	Sputum (Human)	China

CQPMC-Pstu	CP171270.1	99.83%	Unknown	*Pseudomonas stutzeri*	Chromosome	Bronchoalveolar lavage fluid (Human)	China
2-216	CP158049.1	99.83%	100% and 41%	*Pseudomonas aeruginosa*	Chromosome	Chicken meat (Animal)	China
BB1456	LR813085.1	99.83%	—	*Pseudomonas putida*	Chromosome	Wastewater (Environment)	Spain
BB1451	LR813083.1	99.83%	—	*Pseudomonas putida*	Chromosome	Wastewater (Environment)	Spain
ZBX-P12	CP061779.1	99.81%	—	*Pseudomonas aeruginosa*	Chromosome	Urine (Human)	Lebanon
170620603RE	CP043396.1	99.81%	100% and 41%	*Pseudomonas monteilii*	Chromosome	Drainage (Environment)	China
NY4817	CP131921.1	99.81%	100% and 41%	*Pseudomonas kurunegalensis*	Chromosome	Human	China
T75	CP113226.1	99.81%		*Pseudomonadaceae*	Chromosome	Pig (Animal)	China
170918607	CP043395.1	99.81%	100% and 41%	*Pseudomonas monteilii*	Chromosome	Drainage (Environment)	China
ZBX-P23	CP061777.1	99.81%	—	*Pseudomonas aeruginosa*	Chromosome	Unknown (Human)	Lebanon
J5083553	CP166822.1	99.79%	—	*Pseudomonas aeruginosa*	Plasmid	Tracheal aspirate (Human)	Brazil
M61216631	CP166820.1	99.79%	—	*Pseudomonas aeruginosa*	Plasmid	Lombar wound (Human)	Brazil
GD04120	CP104580.1	99.79%	—	*Pseudomonas stutzeri*	Chromosome	Surface swab (Environment)	Pakistan

1334/14	CP035739.1	99.77%	—	*Pseudomonas aeruginosa*	Chromosome	Eye (Human)	Poland
GD03919	CP104582.1	99.77%	—	*Pseudomonas* sp.	Chromosome	Surface swab (Environment)	Pakistan
GD03721	CP104583.1	99.77%	—	*Pseudomonas* sp.	Chromosome	Water (Environment)	Pakistan
PS_234	RHQZ01000017.1	99.77%	—	*Pseudomonas stutzeri*	Unknown	Bedside rail in hospital intensive care (Environment)	Pakistan
ZM21	CP141683.1	99.63%	100% and 41%	*Pseudomonas aeruginosa*	Chromosome	Activated sludge (Environment)	China

"—" showed the upstream region of *tnfxB6-tmexC6D6-toprJ1b* not contained *hp-2*-like gene.

[a] indicated the sequence of *hp-2*-like gene was completely similar to this gene of FJ21PNM24.

Table S3 The information for *tmexC6D6-toprJ1b* and *tmexC6D6-toprJ1b*-like gene clusters of pig origin

Gene	Species	Location of *tmexCD-toprJ*	Source	The positive rate of *tmexCD-toprJ*	Country	Reference
tmexC1D1-toprJ1	Unknown	Unknown	Pork (n=12)	3.41%, 12 *tmexC1D1-toprJ1* screened from 352 retail pork samples	Hunan and Guangdong Province, China	[1]
tmexC1D1-toprJ1	*Klebsiella Pneumoniae*	chromosome	Pork (n=1)	0.85%, 1 *tmexC1D1-toprJ1* screened from 117 pork samples	Jiangsu Province, China	[3]
tmexC3D3-toprJ1b	*Proteus mirabilis*	Chromosome	Feces (n=1)	0.55%, 1 *tmexC3D3-toprJ1b* screened from 182 swine fecal samples in a swine slaughterhouse	Jiangsu Province, China	[4]
tmexC1D1-toprJ1 (n=1)	*K. Pneumoniae*	Plasmid	Pork (n=1)	0.72%, 1 *tmexC1D1-toprJ1* screened from 139 pork samples	Guangdong Province, China	[5]
tmexC1D1-toprJ1 (n=22)<br>*tmexC3D3-toprJ1b* (n=2)	*K. Pneumoniae*<br>*P. mirabilis*	Plasmid<br>Chromosome	Feces (n=15)<br>Blood (n=1)<br>Wastewater (n=1)<br>Carcass (n=7)	7.32%, 24 *tmexC1D1-toprJ1* screened from 328 samples, including feces, blood, wastewater, soil, and carcass, in swine slaughterhouses	Jiangsu Province, China	
tmexC3D3-toprJ1b	*Proteus terrae*	Chromosome	Fece (n=1)	0.57%, 1 *tmexC3D3-toprJ1b* screened from 175 samples, including feces, blood, wastewater, and environment, in a swine slaughterhouse	Jiangsu Province, China	[6]

Reference

1. Lv L, Wan M, Wang C, Gao X, Yang Q, Partridge SR, Wang Y, Zong Z, Doi Y, Shen J, Jia P, Song Q, Zhang Q, Yang J, Huang X, Wang M, Liu JH. Emergence of a Plasmid-Encoded Resistance-Nodulation-Division Efflux Pump Conferring Resistance to Multiple Drugs, Including Tigecycline, in Klebsiella pneumoniae. mBio. 2020;11(2).
2. Wang CZ, Gao X, Tu JY, Lv LC, Pu WX, He XT, Jiao YX, Deng YT, Liu JH. Multiple Copies of Mobile Tigecycline Resistance Efflux Pump Gene Cluster tmexC2D2.2-toprJ2 Identified in Chromosome of Aeromonas spp. Microbiology spectrum. 2022;10(6):e0346822.
3. Sun L, Wang H, Meng N, Wang Z, Li G, Jiao X, Wang J. Distribution and Spread of the Mobilized RND Efflux Pump Gene Cluster tmexCD-toprJ in Klebsiella pneumoniae from Different Sources. Microbiology spectrum. 2023;11(4):e0536422.
4. Wang Q, Peng K, Liu Y, Xiao X, Wang Z, Li R. Characterization of TMexCD3-TOprJ3, an RND-Type Efflux System Conferring Resistance to Tigecycline in Proteus mirabilis, and Its Associated Integrative Conjugative Element. Antimicrobial agents and chemotherapy. 2021;65(7):e0271220.
5. Peng K, Wang Q, Yin Y, Li Y, Liu Y, Wang M, Qin S, Wang Z, Li R. Plasmids Shape the Current Prevalence of tmexCD1-toprJ1 among Klebsiella pneumoniae in Food Production Chains. mSystems. 2021;6(5):e0070221.
6. Peng K, Li Y, Wang Q, Yang P, Wang Z, Li R. Integrative conjugative elements mediate the high prevalence of tmexCD3-toprJ1b in Proteus spp. of animal source. mSystems. 2023;8(5):e0042923.
